# L-Ascorbic Acid and α-Tocopherol Synergistically Triggers Apoptosis Inducing Antileukemic Effects of Arsenic Trioxide *via* Oxidative Stress in Human Acute Promyelocytic Leukemia Cells

**DOI:** 10.3389/fonc.2020.00065

**Published:** 2020-02-21

**Authors:** Radhakrishnan Chandraprabha Vineetha, Sreedharan Hariharan, Abdul Jaleel, Mahesh Chandran, Raveendran Harikumaran Nair

**Affiliations:** ^1^Physiology Research Laboratory, School of Biosciences, Mahatma Gandhi University, Kottayam, India; ^2^Laboratory of Cytogenetics and Molecular Diagnostics, Division of Cancer Research, Regional Cancer Centre, Trivandrum, India; ^3^Proteomics Facility, Rajiv Gandhi Centre for Biotechnology, Trivandrum, India

**Keywords:** acute promyelocytic leukemia, arsenic trioxide, HL-60 cells, L-ascorbic acid, α-tocopherol

## Abstract

Chemosensitization is an effective strategy to overcome the drawbacks of arsenic trioxide (As_2_O_3_) treatment, which may be possible through the use of dietary supplements in combination. The present investigation evaluates the synergistic mechanism of action of vitamins, such as L-ascorbic acid (L-AA) and α-tocopherol (α-TOC) in As_2_O_3_ chemotherapy using human leukemia (HL-60) cells. *In vitro* assays on the cytotoxicity of As_2_O_3_ and vitamins and cellular apoptotic evidences were done; a proteomic investigation with mass spectrometry was also performed. The combination of L-AA and α-TOC potentiates As_2_O_3_ cytotoxicity in HL-60 cells, substantiated by depletion in antioxidant status, mitochondrial transmembrane potential, and inhibition of nuclear factor erythroid 2-related factor 2 and B-cell lymphoma 2 transcription factors. Mass spectrometry results showed decreased expression of proteins regulating cell cycle and translation in cells treated with As_2_O_3_, L-AA, and α-TOC when compared with As_2_O_3_-treated sample. In addition, this combination treatment identified numerous proteins associated with apoptosis and cell stress. HL-60 cells became more prone to As_2_O_3_ on exposure to L-AA and α-TOC, indicating that this combination may be a promising approach to increase the outcome of As_2_O_3_ chemotherapy.

## Introduction

Acute promyelocytic leukemia (APL) is a particular subtype of acute myeloid leukemia (AML) distinguished by blast cell morphology. It attributes about 10–15% of the AML. Generally, it is characterized by the expression of promyelocytic leukemia (PML)/retinoic acid receptor-α (RARα) fusion oncoprotein, a specific chromosome translocation *t*(15,17) due to the fusion between the genes PML and the RARα. This PML/RARα disturbs the cellular signaling of wild-type RARα in myeloid progenitor cells resulting in a differentiation block at the promyelocyte stage (1). The presence of coagulation disorders is an important risk factor for early hemorrhagic death in patients with an APL. This remains the vital cause of death during induction therapy in these patients (2). The treatment for APL with all-trans retinoic acid and anthracycline-based chemotherapy resulted in the cure rates of ~80%. Thus, the combination of all-trans retinoic acid and chemotherapy was considered the standard of care for newly diagnosed APL (3). Moreover, arsenic trioxide (As_2_O_3_) is also highly effective in the treatment of APL.

Over 2,000 years, arsenic compounds were used to treat various malignant and inflammatory diseases in Chinese medicine. There is comprehensive proof specifying that arsenic in the form of As_2_O_3_ shows powerful antitumor effects both *in vitro* and *in vivo*. As_2_O_3_ (TRISENOX) was approved in September 2000 by the United States Food and Drug Administration for the treatment of relapsed and refractory APL (4). The efficacy of arsenic compounds is based on several modes of actions. The rise in reactive oxygen species (ROS) is a significant primary cellular event that happens during the therapy of As_2_O_3_ target cells (5). Excess production of ROS triggers lipid peroxidation, an important mediator of oxidative stress that causes cell injury leading to death of cells (6). Arsenic derivatives are powerful redox modulators targeting the thioredoxin system comprising of thioredoxin, thioredoxin reductase, and nicotinamide adenine dinucleotide phosphate that plays a significant role in controlling intracellular redox reactions, apoptosis, and protecting cells against stress. Compounds that contain arsenic can target and block thioredoxin reductase that may be essential in inducing its proapoptotic impacts (7).

On the other hand, several studies point out that the single agent As_2_O_3_ is ineffectual as expected against APL and other malignant tumors clinically (8). In APL treatment, the elevated incidence of early deaths among patients remains a major issue. Chemosensitization offers an effective strategy to overcome the drawbacks involved in As_2_O_3_ treatment. This can be done using dietary supplements that can be used alone or combined with standard chemotherapy agents (9). A study by Landis-Piwowar et al. (10) reported that pharmacologically safe compounds in combination with standard chemotherapeutic drugs can behave as potent chemosensitizers. Reports from many studies suggested the use of antioxidants to enhance arsenic-trioxide-mediated apoptosis in tumor cells (11). Ascorbic acid/Vitamin C is regarded as an important antioxidant molecule found to enhance As_2_O_3_ toxicity in malignant cells *in vitro* (12), by acting as a prooxidant (13). The redox chemical properties of vitamin C are responsible for its antioxidant as well as potential pro-oxidant activities. α-Tocopherol (α-TOC) is relatively low toxic and can act as an important micronutrient for maintaining the stability between antioxidant and prooxidant reactions in tissues and is the major form of vitamin E found in blood and tissues (14).

One of the essential elements of cellular antioxidant defense mechanisms is the nuclear factor erythroid 2-related factor 2 (Nrf2). Nrf2 produces a cellular defense mechanism by upregulating intracellular antioxidant that detoxifies drugs and environmental pollutants (15). Apoptosis is one of the main processes involved in cell damage mediated by As_2_O_3_. Bcl2 downregulation could lead to cell apoptosis caused by As_2_O_3_. Upregulation of Bcl2 gene expression by Nrf2 plays a significant role in the blockage of apoptosis and cell survival (16). Here, we show that L-ascorbic acid (L-AA) and α-TOC in combination with As_2_O_3_ inhibits the expression of Nrf2 and Bcl2 genes in the APL cell line, HL-60. Overall, the analysis of Nrf2 and Bcl2 messenger RNA (mRNA) expressions and the proteomics-based study on the expression of a group of heat shock proteins and proteins related to oxidative stress provides a better understanding of the synergistic induction of apoptotic cell death by As_2_O_3_ and vitamins.

## Materials and Methods

### Materials

As_2_O_3_, α-TOC, L-AA, Primers [Nrf2, Bcl2, glyceraldehyde 3-phosphate dehydrogenase (GAPDH)], Triton X 100, dichlorofluorescein diacetate, and Rhodamine-123 (R-123) were purchased from Sigma-Aldrich, Bangalore, India. RapiGest SF was purchased from Waters India Pvt Ltd, Bangalore, India. Fetal bovine serum (FBS), antibiotic–antimycotic solution, Roswell Park Memorial Institute-1640 medium, ethidium bromide, and all other chemicals were purchased from Himedia Pvt. Ltd (Mumbai, India). As_2_O_3_ (1 mM) and L-AA (10 mM) were prepared in aqueous solutions and kept as stock solutions. Stock solution of α-TOC (10 mM) was prepared in 0.2% ethanol. These solutions were diluted to working solutions of As_2_O_3_ (10 μM), L-AA (100 μM), and α-TOC (50 μM) on the experiment day before use.

### Cell Culture and Drug Treatment

Human leukemia (HL-60) cells obtained from National Centre for Cell Sciences, Pune, was used in this research. HL-60 cells were grown in Roswell Park Memorial Institute-1640 medium supplemented with 10% FBS and 1% antibiotic–antimycotic solution in a 5% CO_2_ incubator at 37°C in a humidified atmosphere in accordance with the standard procedures (17). Before starting the experiments, the cells were grown in suspension *in vitro*. For studying the synergistic effect of vitamins with As_2_O_3_ on HL-60 cells, the experimental groups were selected as follows: (a) control cells, (b) 10 μM As_2_O_3_, (c) 100 μM L-AA, (d) 50 μM α-TOC, (e) 10 μM As_2_O_3_ and 100 μM L-AA, (**f**) 10 μM As_2_O_3_ and 50 μM α-TOC, and (**g**) 10 μM As_2_O_3_, 100 μM L-AA, and 50 μM α-TOC. For all the experimental groups, the exposure time was 48 h.

### Determination of Antioxidant Enzyme Activities

Reduced glutathione (GSH) was quantified according to the method of Moron et al. (18). GSH was measured by reaction with 5,5′-dithiobis (2-nitrobenzoic acid) to give a product that absorbs at 412 nm. Catalase is a major enzyme catalyzes the decomposition of H_2_O_2_ to water and oxygen. It is found in all living organisms exposed to oxygen. The level of catalase (CAT) was measured by monitoring the disappearance of H_2_O_2_ at 240 nm, according to the method of Sinha (19). Superoxide dismutase was measured spectrophotometrically according to the method of Kakkar et al. (20). It depends on the inhibition of NADH-dependent nitroblue tetrazolium reduction by dismutase.

### Determination of Mitochondrial Membrane Potential

R-123 is a lipophilic, cationic indicator used to study the electrical potential across the inner mitochondrial membrane. After 48 h of incubation, the treated cells were rinsed with phosphate-buffered saline (PBS). R-123 solution (10 μg/ml) was added to the wells and was incubated in the dark for 20–30 min at 37°C. Later, the cells were washed twice with PBS, and the images were taken using a fluorescence microscope (Olympus) at 20× magnification, and the relative fluorescence was also measured in a multiwall plate reader (21).

### Determination of Cellular Calcium Levels

Cells in the dishes were collected by centrifugation after treatment. They were lysed with cell lysis buffer on ice for 30 min, and the supernatant was used to measure the calcium levels by calcium assay kit (Cayman Chemical Company, Ann Arbor, USA) according to the manufacturer's instruction. This assay uses o-cresolphthalein–calcium reaction, where the formation of a vivid purple complex that absorbs between 560 and 590 nm indicates the presence of calcium. The color intensity is directly proportional to the calcium concentration in the sample.

### Intracellular ROS Detection

A cell permeable fluorescent probe, 2′,7′-dichlorofluorescin diacetate was used for determining the intracellular levels of ROS. After 48 h of drug treatment, dichlorofluorescein diacetate (10 μM) was added to the dishes and incubated in dark for 30 min at 37°C. The cells were washed twice with PBS twice, and images were taken on a fluorescent microscope (Olympus) at 20× magnification. The relative fluorescence was also measured by spectrofluorimeter with an excitation wavelength of 480 nm and an emission wavelength of 530 nm (22).

### Determination of Nrf2 and Bcl2 Gene Expression Using Reverse Transcriptase PCR

Total RNA was isolated from HL-60 cells after respective treatments using RNA iso Plus *(TAKARA Bio Inc. Shiga, Japan)* following manufacturer's instructions. The Nrf2 and Bcl2 expression at mRNA level was studied using the isolated RNA. Using Primescript One-Step RT-PCR kit (TAKARA Bio Inc. Shiga, Japan), conventional reverse transcriptase PCR (RT-PCR) was carried out. The primer sequences with reaction conditions used were as follows: Nrf2—forward 5′CCAACACACGGTCCACAGCT3′, reverse 5′TCCGTCGCTGACTGAAGTCCA 3′. Fifteen-minutes initial enzyme activation at 95°C followed by 30 cycles of denaturation (95°C for 10 s), annealing (59°C for 15 s) and extension (72°C for 20 s) with a final extension of 72°C for 5 min. Bcl2—forward 5′ GTTTGATTTCTCCTGGCTGTCTC 3′, reverse 5′ GACCTTTTGCATATTTGTTTGG 3′. The reaction consists of a first cycle at 10 min for 95°C followed by 28 cycles of denaturation (94°C for 30 s), annealing (60°C for 30 s), extension (72°C for 90 s), and a final extension of 72°C for 5 min; GAPDH—forward 5′ GTGAAGGTCGGAGTCAACG 3′, reverse 5′ TGAGGTCAATGAAGGGGTC 3′. The reaction consists of an initial cycle of 50°C for 30 min and 94°C for 2 min followed by 40 cycles of denaturation (94°C for 30 s), annealing (55°C for 30 s), extension (72°C for 1 min), and a final extension of 72°C for 5 min. One percent agarose gels containing 0.05 μg/ml ethidium bromide was used for electrophoresing the PCR products. Using a 100-bp DNA ladder, the bands were identified according to their product size. The gel image was viewed and recorded with a gel documentation system, and the band densities were quantified using Quantity One®1-D Image analysis software of Bio-rad Image Analysis Systems (CA, United States) by densitometric analysis. The relative expression of the samples were compared and normalized with their corresponding GAPDH expressions.

### Proteomic Profiling

#### Protein Sample Preparation

HL-60 cells were washed twice with PBS and centrifuged at 9,000 rpm to eliminate traces of FBS. The cell lysate was prepared in 0.1% acid-labile detergent RapiGest SF in 25 mM ammonium bicarbonate buffer. Protein quantification was performed by Bradford assay (23). Using in-solution trypsin digestion, peptide was produced for each sample (100 μg of protein). The digested peptide solutions were centrifuged at 20,817 × *g* for 12 min. The supernatant was stored at −20°C before liquid chromatography tandem mass spectrometry (LC-MS/MS) analysis.

#### Liquid Chromatography Tandem Mass Spectrometry

The tryptic peptides were separated using a nanoACQUITY UPLC® chromatographic system (Waters, Manchester, United Kingdom). Instrument control and data processing was done with MassLynx4.1 SCN781 software. The peptides were separated by reversed-phase chromatography. MS analysis of eluting peptides was carried out on a SYNAPT® G2 High-Definition MS™ System (HDMS^E^ System (Waters). All analyses were carried out in positive mode electrospray ionization with a NanoLockSpray™ source.

#### Mass Spectrometry Data Analysis

The acquired ion mobility-enhanced MSE spectra was analyzed using Progenesis QI for Proteomics V3.0 (Non-Linear Dynamics, Waters) for protein identification and for the label-free relative protein quantification. Data processing includes lock mass correction post-acquisition. Processing parameters for Progenesis were as follows: noise reduction thresholds for low energy scan ion, 150 counts; high energy scan ion, 30 counts. The protein identifications were acquired by searching against the human protein database downloaded from UniProt. The protein false positive rate was set to 4% during database search. The parameters for protein identification was made in such a way that a peptide was required to have at least one fragment ion match, a protein was required to have at least three fragment ion matches, and a protein was required to have at least one peptide match for identification. Cysteine carbamidomethylation was chosen as a fixed modification, and oxidation of methionine was chosen as variable modification. Trypsin was selected as the enzyme used with a specificity of one missed cleavage. Data sets were analyzed and quantified using relative quantitation using Hi–N algorithm, which resolve peptide conflicts and uses the average intensity of the three most abundant unique peptides for a protein. Moreover, simply a fold change >50% difference (ratio of either <0.50 or >1.50) was believed to be indicative of considerably distorted levels of expression.

### Statistical Analysis

Data were obtained from repeated experiments (*n* = 6), and the results were presented as mean ± standard deviations of the control and treated cells. Statistical analysis of the results was performed using SPSS/PC+version16 (SPSS Inc. Chicago, IL, USA). One-way ANOVA with least significant difference *post-hoc* multiple comparison tests was employed to determine significant difference among groups. *P* < 0.05 was considered as statistically significant.

## Results

### GSH, CAT, and SOD Levels in HL-60 Cells

**Figure 1:**
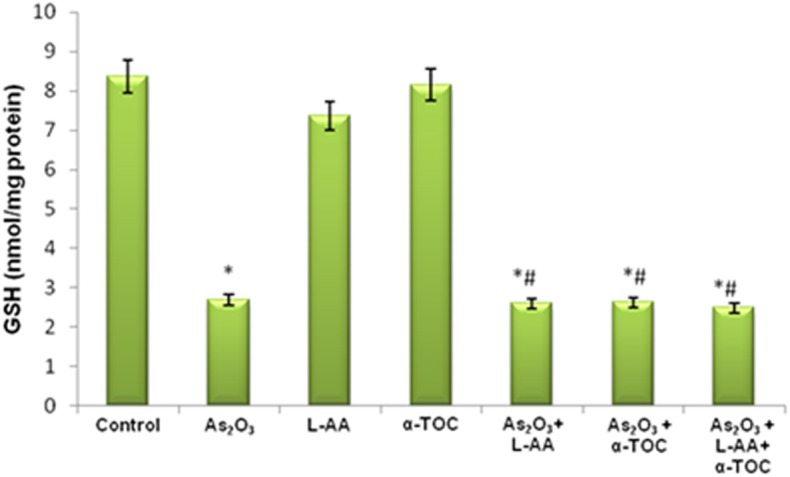
Effect of arsenic trioxide and vitamins on reduced glutathione (GSH) activity. Data represented as mean ± SD, asterisk represents comparisons with control cells (*p* < 0.05), and number sign represents comparisons with As_2_O_3_-treated group (*p* < 0.05).

**Figure 2:**
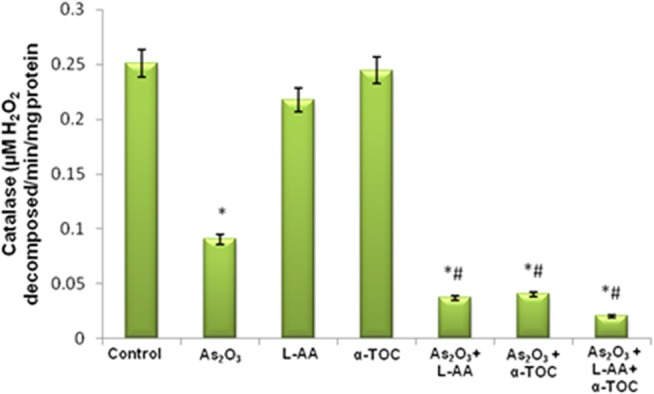
Effect of arsenic trioxide and vitamins on catalase (CAT) activity. Data represented as mean ± SD, asterisk represents comparisons with control cells (*p* < 0.05), and number sign represents comparisons with As_2_O_3_-treated group (*p* < 0.05).

**Figure 3:**
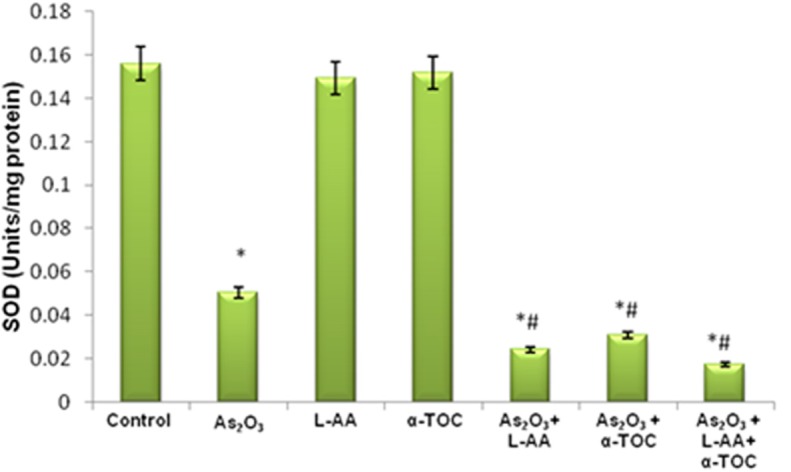
Effect of arsenic trioxide and vitamins on superoxide dismutase (SOD) activity. Data represented as mean ± SD, asterisk represents comparisons with control cells (*p* < 0.05), and number sign represents comparisons with As_2_O_3_-treated group (*p* < 0.05).

The growth inhibitory and apoptotic effect of As_2_O_3_ inversely correlates with the GSH content of HL-60 cells. A significant inhibition in the GSH levels was observed in As_2_O_3_-treated cells in comparison with control cells. In addition, a considerable decline in the GSH level was found in the cells coincubated with As_2_O_3_ and vitamins (Figure 1). Likewise, a significant reduction (*P* < 0.05) in the levels of antiperoxidative enzymes, CAT, and superoxide dismutase (SOD) was also found in the cells treated with As_2_O_3_ compared with the untreated cells. Our results suggested a significant inhibition in the enzyme levels in the experimental group with 10 μM As_2_O_3_, 100 μM L-AA, and 50 μM α-TOC (Figures 2, 3).

### L-Ascorbic Acid and α-Tocopherol With Arsenic Trioxide Modifies the Mitochondrial Membrane Potential in HL-60 Cells

**Figure 4:**
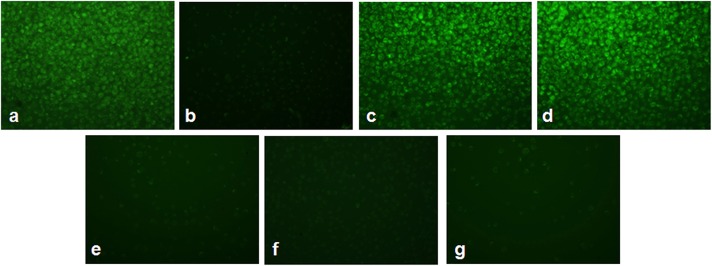
Detection of mitochondrial membrane potential using Rhodamine-123 in HL-60 cells. **(a)** Control cells, **(b)** 10 μM Arsenic trioxide, **(c)** 100 μM L-ascorbic acid, **(d)** 50 μM α-tocopherol, **(e)** 10 μM arsenic trioxide and 100 μM L-ascorbic acid, **(f)** 10 μM arsenic trioxide and 50 μM α-tocopherol, and **(g)** 10 μM arsenic trioxide, 100 μM L-ascorbic acid, and 50 μM α-tocopherol.

**Figure 5:**
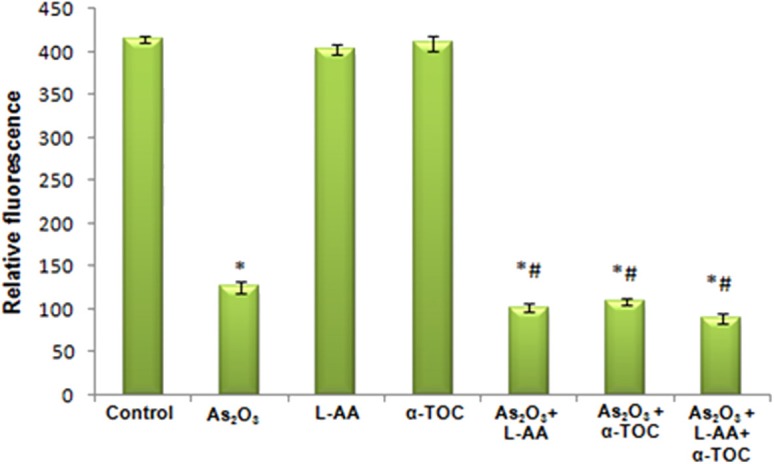
Determination of relative fluorescence as a measure of mitochondrial membrane potential. The values are represented as mean ± SD, asterisk represents comparisons with control cells (*P* < 0.05), and number sign represents comparisons with As_2_O_3_-treated group (*P* < 0.05).

Figure 4 shows the alterations in mitochondrial membrane potential in As_2_O_3_-treated groups and vitamin cotreated groups. A considerable reduction in the mitochondrial membrane potential was observed on treatment with 10 μM As_2_O_3_ when compared with the control group as represented by Figure 4b. HL-60 cells treated only with L-AA and with α-TOC showed mitochondrial membrane potential similar to that of the untreated cells (Figures 4c,d). Cotreatment of L-AA and with α-TOC (Figure 4g) exhibits a considerable decrease in mitochondrial membrane potential as visible from the low fluorescence in contrary to cells treated with As_2_O_3_ alone, which was supported by fluorimetric analysis data (Figure 5).

### Cotreatment of L-Ascorbic Acid and α-Tocopherol With As_2_O_3_ Alters the Calcium Production in HL-60 Cells

**Figure 6:**
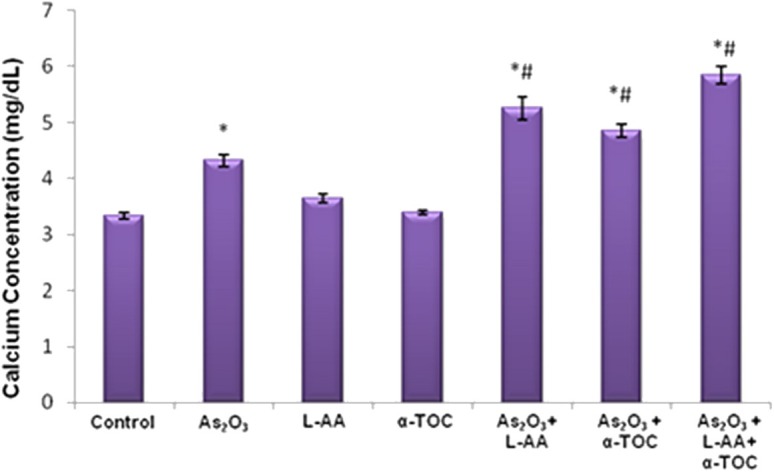
Effect of arsenic trioxide with L-ascorbic acid and α-tocopherol on calcium level in HL-60 cells. The values are represented as mean ± SD, asterisk represents comparisons with control cells (*P* < 0.05), and number sign represents comparisons with As_2_O_3_-treated group (*P* < 0.05).

The results showed a considerable elevation in the calcium concentration in As_2_O_3_-treated group, As_2_O_3_ + L-AA and As_2_O_3_ + α-TOC. Moreover, the combination of L-AA and α-TOC with As_2_O_3_ significantly elevated the level of calcium in HL-60 cells (Figure 6).

### As_2_O_3_ With Vitamins Increases the ROS Production in HL-60 Cells

**Figure 7:**
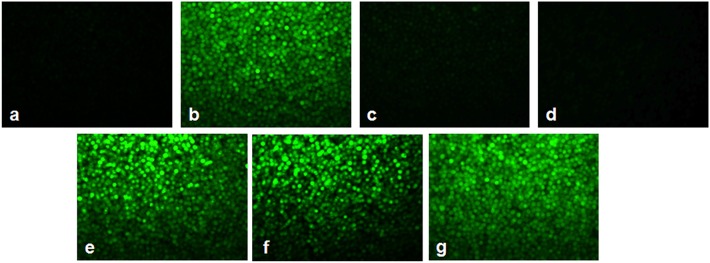
Effect of L-ascorbic acid and α-tocopherol on intracellular reactive oxygen species (ROS) production. **(a)** Control cells, **(b)** 10 μM arsenic trioxide, **(c)** 100 μM L-ascorbic acid, **(d)** 50 μM α-tocopherol, **(e)** 10 μM arsenic trioxide and 100 μM L-ascorbic acid, **(f)** 10 μM arsenic trioxide and 50 μM α-tocopherol, and **(g)** 10 μM arsenic trioxide, 100 μM L-ascorbic acid, and 50 μM α-tocopherol.

**Figure 8:**
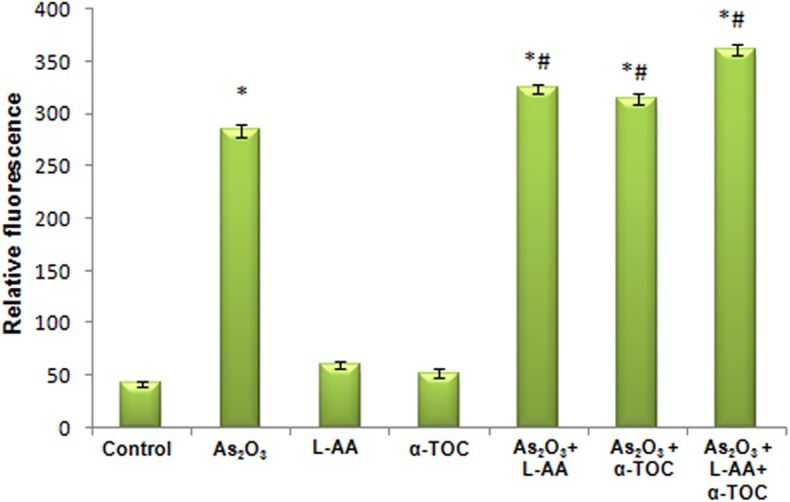
Amount of reactive oxygen species release measured as fluorescence. The values are represented as mean ± SD, asterisk represents comparisons with control cells (*P* < 0.05), and number sign represents comparisons with As_2_O_3_-treated group (*P* < 0.05).

Figures 7, 8 showed the outcome of ROS production in all the experimental groups. A significant elevation in the green fluorescence was observed in the APL cells treated with 10 μM As_2_O_3_ in comparison with the control cells. Figure 7g showed that the combination treatment of As_2_O_3_ with L-AA and α-TOC demonstrated a rise in the green fluorescence which means increased ROS production.

### Detection of Nrf2 and Bcl2 Gene Expression in HL-60 Cells

**Figure 9:**
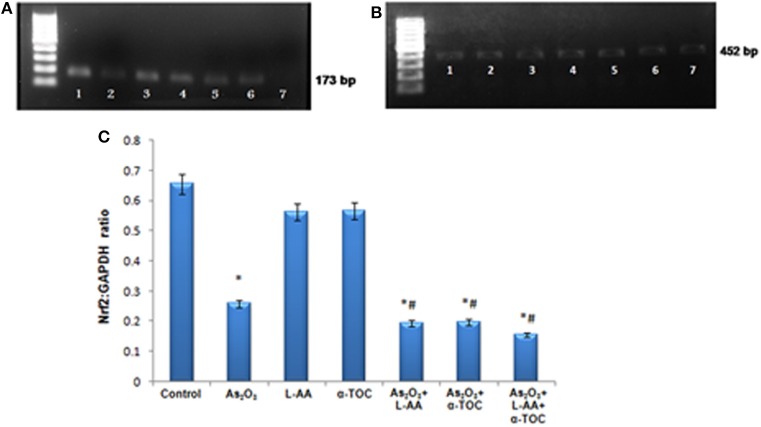
**(A)** Analysis of Nrf2 messenger RNA (mRNA) expression using conventional reverse transcriptase PCR (RT-PCR). **(B)** RT-PCR of glyceraldehyde 3-phosphate dehydrogenase (GAPDH) from the samples taken as the internal control. Lane (1) control cells, (2) arsenic trioxide, (3) L-ascorbic acid, (4) α-tocopherol, (5) arsenic trioxide and L-ascorbic acid, (6) arsenic trioxide and α-tocopherol, and (7) arsenic trioxide, L-ascorbic acid, and α-tocopherol. **(C)** Quantitative densitometric analysis of Nrf2 expression. The values are represented as mean ± SD, asterisk represents comparisons with control cells (*P* < 0.05), and number sign represents comparisons with As_2_O_3_-treated group (*P* < 0.05).

**Figure 10:**
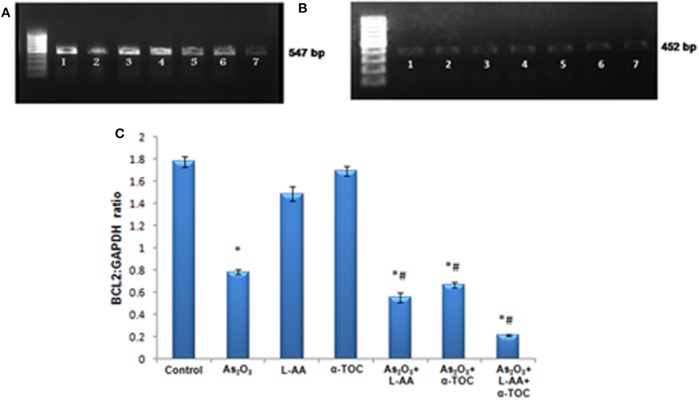
**(A)** Analysis of Bcl2 messenger RNA (mRNA) expression using conventional reverse transcriptase PCR (RT-PCR). **(B)** RT-PCR of glyceraldehyde 3-phosphate dehydrogenase (GAPDH) from the samples taken as the internal control. Lane (1) control cells, (2) arsenic trioxide, (3) L-ascorbic acid, (4) α-tocopherol, (5) arsenic trioxide and L-ascorbic acid, (6) arsenic trioxide and α-tocopherol and (7) arsenic trioxide, L-ascorbic acid, and α-tocopherol. **(C)** Quantitative densitometric analysis of Bcl2 expression. The values are represented as mean ± SD, asterisk represents comparisons with control cells (*P* < 0.05), and number sign represents comparisons with As_2_O_3_-treated group (*P* < 0.05).

RT-PCR identifies the mRNA expressions of Nrf2 and Bcl2 genes in HL-60 cells. The Nrf2 gene expression is basal in the control cells (Figure 9A), while cells treated with As_2_O_3_ exhibits a noticeable reduction in the expression of Nrf2 transcription factor in contrast with the control cells. No significant change was observed in Nrf2 gene expression in the cells treated with L-AA and α-TOC. Very faint bands were observed in the experimental groups treated with a combination of As_2_O_3_ and L-AA and As_2_O_3_ and α-TOC. A marked decrease in the Nrf2 gene expression was observed in the group treated with As_2_O_3_, L-AA, and α-TOC. The GAPDH expressions in the respective samples are shown in Figure 9B. Detection of Bcl2 gene expression with total RNA showed a faint band in the As_2_O_3_-treated group (Figure 10A). In the untreated cells and cells treated with L-AA and α-TOC, the band intensity was almost similar. Very faint bands were seen in the experimental groups treated with As_2_O_3_ and L-AA and As_2_O_3_ and α-TOC. No band was found in the treatment group with As_2_O_3_, L-AA, and α-TOC. Figure 10B showed the respective band intensities of GAPDH. The Nrf2 gene expression was dependable with the Bcl2 gene expression, with the combination treatment group having As_2_O_3_, L-AA, and α-TOC exhibits the least concentration (Figures 9C, 10C).

### Analysis of Differentially Expressed Protein Profiles Between Apoptotic and Normal HL-60 Cells

**Figure 11:**
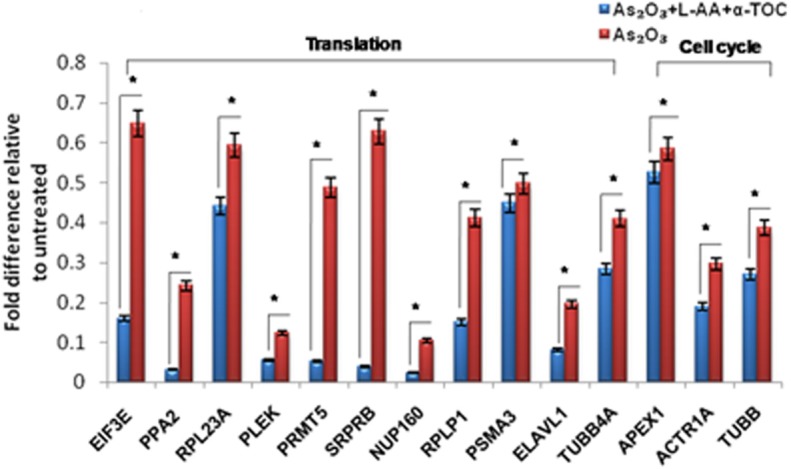
Liquid chromatography mass spectrometry (LC-MS) analysis showed expression of proteins regulating translation and cell cycle. The values are represented as mean ± SD, *P < 0.05 indicates As_2_O_3_ vs. As_2_O_3_ + L-AA + α-TOC. EIF3E, eukaryotic translation initiation factor 3 subunit E; PPA2, inorganic pyrophosphatase 2; RPL23A, 60S ribosomal protein L23a; PLEK, Pleckstrin; PRMT5, protein arginine *N*-methyltransferase 5; SRPRB, signal recognition particle receptor subunit beta; NUP160, nuclear pore complex protein; RPLP1, 60S acidic ribosomal protein P1; PSMA3, proteasome subunit alpha type-3; ELAVL1, ELAV-like protein 1; TUBB4A, tubulin beta-4A chain; APEX1, DNA-(apurinic or apyrimidinic site) lyase; ACTR1A, alpha-centractin; TUBB, tubulin beta chain.

**Figure 12:**
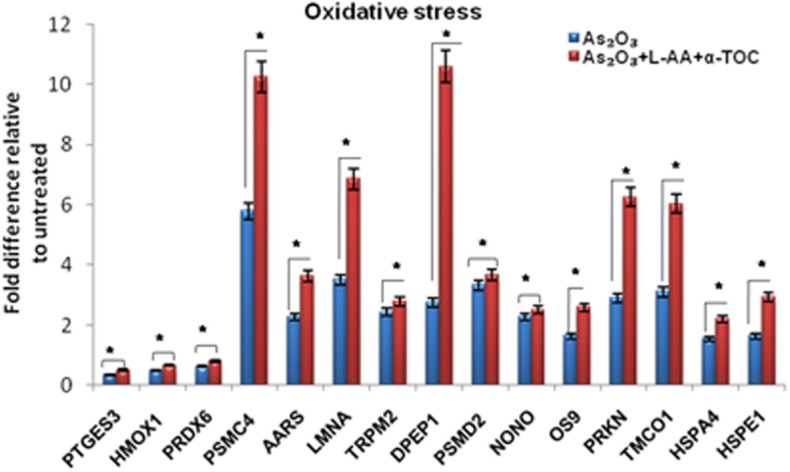
Liquid chromatography mass spectrometry (LC-MS) analysis showed the expression of stress-related proteins. The values are represented as mean ± SD, *P < 0.05 indicates As_2_O_3_ vs. As_2_O_3_ + L-AA + α-TOC. PTGES3, prostaglandin E synthase 3; HMOX1, heme oxygenase-1; PRDX6, peroxiredoxin-6; PSMC4, 26S protease regulatory subunit 6B; AARS, alanine–tRNA ligase; LMNA, prelamin-A/C; TRPM2, transient receptor potential cation channel subfamily M member 2; DPEP1, dipeptidase 1; PSMD2, 26S proteasome non-ATPase regulatory subunit 2; NONO, non-POU domain-containing octamer-binding protein; OS9, protein OS-9; PRKN, E3 ubiquitin-protein ligase parkin; TMCO1, calcium load-activated calcium channel; HSPA4, heat shock 70 kDa protein 4; HSPE1, 10 kDa heat shock protein.

**Figure 13:**
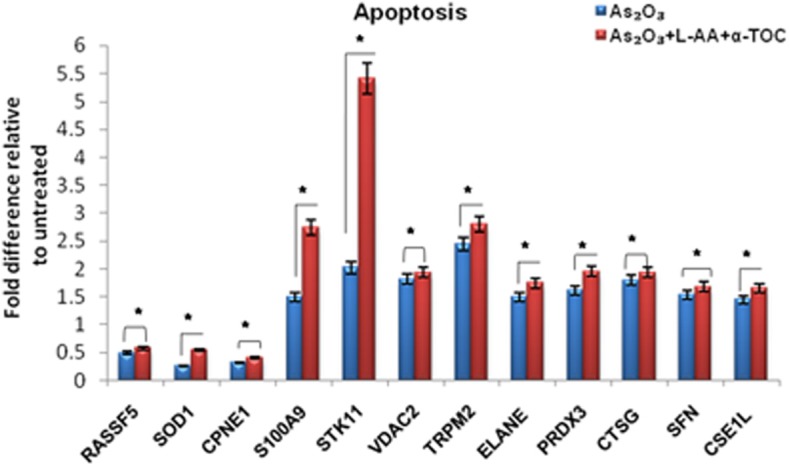
Liquid chromatography mass spectrometry (LC-MS) analysis showed the expression of apoptosis-related proteins. The values are represented as mean ± SD, *P < 0.05 indicates As_2_O_3_ vs. As_2_O_3_ + L-AA + α-TOC. RASSF5, Ras association domain-containing protein 5; SOD1, superoxide dismutase [Cu–Zn]; CPNE1, copine-1; S100A9, protein S100-A9; STK11, serine/threonine-protein kinase; VDAC2, voltage-dependent anion-selective channel protein; TRPM2, transient receptor potential cation channel subfamily M member 2; ELANE, neutrophil elastase; PRDX3, thioredoxin-dependent peroxide reductase; CTSG, cathepsin G; SFN, 14–3–3 protein sigma; CSE1L, exportin-2.

To identify the molecular mechanism behind the combination effect of As_2_O_3_ treatment with vitamins, we performed LC-MS analysis from HL-60 cell samples of control, As_2_O_3_ treated, and the combination of As_2_O_3_ with vitamins. Protein expression profiles revealed that HL-60 cells after treatment with a combination of 10 μM As_2_O_3_, 100 μM L-AA, and 50 μM α-TOC showed decreased expression of proteins regulating cell cycle and translation in comparison with samples treated with As_2_O_3_ alone (Figure 11). The treatment condition used, 10 μM As_2_O_3_ for 48 h, would lead to significant apoptosis in the HL-60 cells, hence elucidating the number of apoptosis and cell stress related protein hits. This was further upregulated on combination treatment of As_2_O_3_ with L-AA and α-TOC (Figures 12, 13). Our data identify several common classes of compounds that altered with apoptotic agents. Moreover, As_2_O_3_ has been found to completely inhibit (fold change infinity) the expression of some of the proteins related to cell cycle, cell division (CDK2, UBE2I), and translation (NUTF2).

Interestingly, numerous proteins changed in response to cotreatment of As_2_O_3_ with L-AA and α-TOC. Several proteins associated with apoptosis and cell stress were recognized on exposure to this combination treatment. Three proteins, BNIP3, PDCD5, and Nek9, were also identified (Table 1). Besides the considerable upregulation of histones, the combination of As_2_O_3_ and vitamins also leads to the downregulation of several important proteins involved in cell cycle, transcription, and translation.

**Table 1:** Unique proteins identified.

**Accession number**	**Protein name**	**Function**
**Unique proteins identified in untreated cells**		
O14737	2′-deoxynucleoside 5′-phosphate N-hydrolase 1 (DNPH1)	Cell proliferation
Q9Y281	HLA class I histocompatibility antigen, alpha chain E (HLA-E)	Immunity
P62993	Cyclin-dependent kinase 2 (CDK2)	Cell cycle, cell division, DNA damage and repair
Q8TD19	SUMO-conjugating enzyme UBC9 (UBE2I)	Cell cycle and cell division
P24941	Nuclear transport factor 2 (NUTF2)	mRNA transport, protein transport
Q12769	Vesicle-associated membrane protein 8 (VAMP8)	Autophagy and protein transport
O60841	60S ribosomal protein L26 (RPL26)	Translation
**Unique proteins identified in cells cotreated with arsenic trioxide and vitamins**		
O60238	BCL2/adenovirus E1B 19 KDa protein-interacting protein 3-like (BNIP3L)	Apoptosis
O14737	Programmed cell death protein 5 (PDCD5)	Apoptosis
Q8TD19	Serine/threonine-protein kinase Nek9 (NEK9)	Cell division and mitotic nuclear envelope disassembly

## Discussion

As_2_O_3_ was previously stated to be cytotoxic to different mammalian cancer cell lines (24). Reports from the earlier studies mentioned that As_2_O_3_ circulate all the way through cell membrane into the cytoplasm and generate cytotoxic effect due to oxidative stress (25). It also induces cell death in a range of leukemic cell lines like APL, AML, and chronic myeloid leukemia in addition to solid tumor cells (26). Leukemic cells have shown to be more liable and clinically significant than the other cells (27). The present research evaluated whether the L-AA and α-TOC could increase the sensitivity of As_2_O_3_ chemotherapy. Treatment with ascorbic acid alone was not cytotoxic, implying that L-AA and α-TOC have the prospective to operate as a secure and efficient chemosensitizing agent in As_2_O_3_-based therapy. Exposure of HL-60 cells with L-AA and α-TOC was linked with a decline in the antioxidant levels, an increase in the intracellular ROS and calcium concentrations, a decrease in mitochondrial membrane potential, and downregulation of Nrf2 and Bcl2 resulting in apoptotic cell death. Remarkably, the combined effect of As_2_O_3_ with L-AA and α-TOC was particular for leukemic cells, as no apoptotic effect was found in normal cells (28, 29).

Reports indicated that antioxidant enzymes are the first line of cellular defense that obstructs cellular constituents from oxidative injury. GSH is a powerful nucleophile decisive for cellular defensive actions, such as detoxification of the generated ROS. The selectivity of vitamin C in killing cancer cells is due to its accumulation inside the cells leading to intracellular GSH depletion. GSH depletion showed the way to harm cellular defense and directed to oxidative damage (30). SOD and CAT cooperatively act as key enzymes in the elimination of ROS. The assessment of innate antioxidant enzymes like GSH, CAT, as well as SOD in the cells treated with L-AA, α-TOC, and As_2_O_3_ was found to be inhibited when compared with the untreated cells.

Oxidative stress has been suggested to take part as a key function in the process of apoptosis induced by As_2_O_3_ (31). It inhibits the activity of calcium-ATPase and leads to altered levels of calcium in the cells. Earlier trials proposed the importance of calcium concentration as one of the major signals of apoptosis (32). In the present study, an elevation in the calcium level was found in the combination treatment group containing As_2_O_3_ and vitamins than As_2_O_3_ alone. Intracellular calcium release plays a part in eliciting ROS production, which is an indicator of oxidative stress (33). Oxidative stress hinders the activity of calcium-ATPase causing changes in the calcium level. Excess production of free radicals also enhances the cytosolic calcium concentration that stimulates endonucleases to facilitate DNA degradation and eventually directs to cell death. As_2_O_3_ exhibits a wide spectrum of antileukemic activity through intracellular ROS production. Elevation in the ROS levels lead to the collapse of the mitochondrial trans-membrane potential (ΔΨm), an important marker for the mitochondrial involvement in apoptosis. The dissipation of ΔΨm shows the way to open mitochondrial permeability transition pore along with the release of cytochrome c resulting in apoptosis (31, 34). Usually, cancer cells are characterized by overproduction of ROS, oxidative challenge, and abnormally elevated antioxidant levels to stabilize it. However, normal cells produce ROS at a lower rate compared to cancer cells and are more resistant to exogenously increased oxidative stress (35). Studies reported that cells exhibiting well-established intracellular antioxidant defense machineries, such as HL-60 cells have shown to be challenging to As_2_O_3_ (36). Usually, cancer cells are characterized by overproduction of ROS, oxidative challenge, and abnormally elevated antioxidant levels to stabilize it. However, normal cells produce ROS at a lower rate compared to cancer cells and are more resistant to exogenously increased oxidative stress (35). It was found that the combined treatment with L-AA and α-TOC renders the leukemic cells more susceptible to As_2_O_3_ as indicated by the lower level antioxidants like GSH, CAT, and SOD and decline in the Nrf2 and Bcl2 mRNA expression when compared with untreated cells and cells treated with As_2_O_3_ alone. Excess generation of free radicals and elevated cytosolic calcium concentration triggers endonuclease that causes DNA degradation and finally leads to cell death.

Although the exact mechanism is not known, this cell-based assay provides evidence that Nrf2 and Bcl2 genes might play critical roles in the synergistic effects of vitamins with As_2_O_3_. Nrf2 attaches to the promoter sequence “antioxidant responsive element” causing the coordinated upregulation of detoxification, cytoprotection, and antioxidant genes driven by antioxidant responsive element (37). It elicits a cellular defense system by upregulating intracellular antioxidants that detoxify drugs (38). We demonstrated that L-AA and α-TOC mediated sensitization of As_2_O_3_ therapy relies on its ability to suppress the Nrf2 gene expression. Thus, downregulation of Nrf2 expression profoundly decreased the expression of key antioxidant enzymes and drug detoxification systems in leukemic cells. For investigating the apoptotic mechanism, we evaluated the effects of combining L-AA and α-TOC on Bcl2 expression. Bcl2 family proteins are key members in mitochondria-mediated intrinsic apoptosis (39). Bcl2 plays an important part for maintaining the normal cell cycle and can hinder apoptosis, thereby acting as a controller of apoptosis. Numerous reports revealed that Bcl2 directs the cell death by hindering the release of mitochondrial cytochrome C release, impeding caspase activity, and regulating the permeability of mitochondrial membrane (40). The present study demonstrated that when the cells were treated with As_2_O_3_, Bcl2 expression was downregulated and was completely inhibited on combined treatment with L-AA, α-TOC, and As_2_O_3_. Studies established that Nrf2 can upregulate antiapoptotic factor Bcl2 and has the ability to act as a vital part in controlling apoptosis (16). Conversely, persistent inactivation of Nrf2 directs the downregulation of antiapoptotic protein Bcl2 causing increased cell death and apoptosis in leukemic cells. At this point, we confirmed that the exposure of HL-60 cells to As_2_O_3_ and vitamins leads to Nrf2 inhibition. Changes in Nrf2 lead to altered Bcl2 expression as well as cellular apoptosis.

Higher concentration of α-TOC results in subsequent oxidative stress and elevated levels of α-TOC radicals that can initiate the process of lipid peroxidation (41). When antioxidant networks are balanced, this prooxidant action of α-TOC radicals is inhibited by coantioxidants, which can reduce the radical back to α-TOC. On the other hand, the prooxidant behavior of ascorbic acid is due to the transition-metal-induced redox cycling and mixed function cosubstrate activity. The production of reductone type products from ascorbic acid and the formation of H_2_O_2_ as a result of ascorbic acid autooxidation reactions contribute to its prooxidant activity. Pharmacological doses of L-AA were shown to reduce proliferation in cancer cells, and the potential mechanism is through the chemical reactions that generates hydrogen peroxide (42). Vitamin C has the capacity to generate α-TOC from α-TOC radical and thereby play a role in maintaining the antioxidant and prooxidant activities of α-TOC.

The proteomic approach was used to monitor a variety of proteins selectively regulated by As_2_O_3_ and vitamins. Other stress-inducible proteins, such as heat shock proteins were upregulated upon treatment with As_2_O_3_ and the combination of L-AA and α-TOC. Our data showed that As_2_O_3_ treatment gave rise to considerable downregulation of heterogeneous nuclear ribonucleoproteins, ribosomal proteins, translation initiation, and elongation proteins. Cotreatment of vitamins with As_2_O_3_ also results in further downregulation of these proteins when compared to As_2_O_3_ treatment. In addition, treatment with As_2_O_3_ gave rise to marked alterations in the expression levels of a number of major enzymes that play significant roles in different cellular pathways. The main protease engaged in the cleavage of PML-RARα fusion protein is neutrophil elastase (ELANE), which is associated with APL (43). We found that ELANE was upregulated on As_2_O_3_ treatment and was further upregulated on cotreatment with L-AA and α-TOC. Cathepsin D (CTSG) is a lysosomal aspartic protease that acts as an important cell death mediator (44). The upregulation of CTSG may also contribute to the cytotoxic effect of As_2_O_3_. Another enzyme, heme oxygenase-1, an Nrf2 target gene, was substantially upregulated upon treatment with As_2_O_3_ (37), which accounts for the generation of ROS. Peroxiredoxins are a family of ubiquitous thioredox-independent peroxidases that play an important role during ROS-dependent signaling, including cell proliferation and apoptosis (45). Overexpression of PRDXs has been found in various kinds of cancers and contributes to chemotherapy resistance (35). Peroxiredoxin proteins have uneven expression levels in leukemia, suggestive of inconsistency in the functional implication depending on the cellular environment. Moreover, our findings suggest that CAT and SOD, but not PRDX family members, play an important role in determining the cellular susceptibility to As_2_O_3_ and vitamins to HL-60 cells. Over all, it was observed that the combination of vitamins with As_2_O_3_ was more able to alter the protein expression in HL-60 cells than with As_2_O_3_ alone. The complex actions of drugs on cells, such as cellular stress and activation of apoptotic pathway, proteasome turnover changes, and cytoskeletal adjustments add to the complex proteomic outcomes.

However, the administration of As_2_O_3_ is a topic of concern in the clinical community, as it may cause several detrimental side effects frequently connected with cardiotoxicity (46) and hepatotoxicity (47). Our findings suggest that the combination of As_2_O_3_ and antioxidant vitamins like L-AA and α-TOC may be particularly efficient in APL cells.

Taken together, we provided evidence confirming that the combination of vitamins with As_2_O_3_ was more able to modify the protein expression in APL cells than with As_2_O_3_ alone. Proteomic analysis revealed that the combination of As_2_O_3_ with vitamins can alter a number of proteins related to the translation, cell cycle, oxidative stress, and apoptotic pathways that adds to their effectiveness in APL treatment. In summary, our study provides the fact that HL-60 cells became more vulnerable to As_2_O_3_ in the presence of L-AA and α-TOC, indicating that this combination may be a hopeful approach to increase the outcome of As_2_O_3_ in the treatment of APL.

## Data Availability Statement

The mass spectrometry proteomics data have been deposited to the ProteomeXchange Consortium via the PRIDE [1] partner repository with the dataset identifier PXD016771 and 10.6019/PXD016771. This data can be found here: http://www.ebi.ac.uk/pride/archive/projects/PXD016771

## Author Contributions

RV designed and carried out the experiments, analyzed the results, and prepared the manuscript. SH provides the facility and guidance in cell line study. MC conducted the LC/MS analysis, and AJ interpreted the data. RN contributed to the data acquisition and its interpretation, and drafted the manuscript for significant intellectual content.

### Conflict of Interest

The authors declare that the research was conducted in the absence of any commercial or financial relationships that could be construed as a potential conflict of interest.
